# Multiple Evolutionary Selections Involved in Synonymous Codon Usages in the *Streptococcus agalactiae* Genome

**DOI:** 10.3390/ijms17030277

**Published:** 2016-02-24

**Authors:** Yan-Ping Ma, Hao Ke, Zhi-Ling Liang, Zhen-Xing Liu, Le Hao, Jiang-Yao Ma, Yu-Gu Li

**Affiliations:** 1 College of Veterinary Medicine, South China Agricultural University, Guangzhou 510642, China; mayanping2292@163.com; 2 Guangdong Open Laboratory of Veterinary Public Health, Guangdong Laboratory for Animal Diseases, Institute of Animal Health, Guangdong Academy of Agricultural Sciences, Guangzhou 510640, China; snailt@126.com (Z.-L.L.); liuzhenxing81@126.com (Z.-X.L.); corallita@sina.com (L.H.); freedom77@21cn.com (J.-Y.M.)

**Keywords:** *Streptococcus agalactiae*, codon usage bias, multiple evolutionary selections, evolution

## Abstract

*Streptococcus agalactiae* is an important human and animal pathogen. To better understand the genetic features and evolution of *S. agalactiae*, multiple factors influencing synonymous codon usage patterns in *S. agalactiae* were analyzed in this study. A- and U-ending rich codons were used in *S. agalactiae* function genes through the overall codon usage analysis, indicating that Adenine (A)/Thymine (T) compositional constraints might contribute an important role to the synonymous codon usage pattern. The GC3% against the effective number of codon (ENC) value suggested that translational selection was the important factor for codon bias in the microorganism. Principal component analysis (PCA) showed that (i) mutational pressure was the most important factor in shaping codon usage of all open reading frames (ORFs) in the *S. agalactiae* genome; (ii) strand specific mutational bias was not capable of influencing the codon usage bias in the leading and lagging strands; and (iii) gene length was not the important factor in synonymous codon usage pattern in this organism. Additionally, the high correlation between tRNA adaptation index (tAI) value and codon adaptation index (CAI), frequency of optimal codons (Fop) value, reinforced the role of natural selection for efficient translation in *S. agalactiae.* Comparison of synonymous codon usage pattern between *S. agalactiae* and susceptible hosts (human and tilapia) showed that synonymous codon usage of *S. agalactiae* was independent of the synonymous codon usage of susceptible hosts. The study of codon usage in *S. agalactiae* may provide evidence about the molecular evolution of the bacterium and a greater understanding of evolutionary relationships between *S. agalactiae* and its hosts.

## 1. Introduction

Synonymous codon usage patterns have great theoretical significance as well as practical values when studying molecular evolution. Synonymous codons are not employed in equal and random frequencies in the 61 codons (excluding three stop codons). Synonymous codon usage patterns often represent additional and essential genetic characteristics evolutionally developed by most life forms. Therefore, synonymous codon usage bias analysis for open reading frames (ORFs) have an important role in underlying mechanisms of synonymous codon usage and gene evolution analysis [1]. Many researchers reported that synonymous codon usage bias existed in a wide range of organisms including bacteria, fungi, parasites, animals and humans [2,3,4,5,6]. Variations in codon usage have been attributed to various determinants such as compositional constraints, selection pressure for translational efficiency, gene expression level, abundance of transfer RNA (tRNA), replicational and transcriptional selection, codon-anticodon interactions, protein secondary structure, gene length, and even hydropathy of proteins [4,7,8,9,10,11,12].

*Streptococcus agalactiae* (*S. agalactiae*) is an important human and animal pathogen, and infection can result in pneumonia, sepsis, meningitis. This bacterium has recently been an important pathogen in fish farms worldwide [13,14,15,16,17]. In tilapia, *S. agalactiae* infection causes disorientation, intracranial edema, hemorrhagic panopthalmitis, exophthalmia, and corneal opacity [18,19,20].

The complete genomes of five *S. agalactiae* strains have been sequenced and these databases can facilitate genetic evolution and pathogenicity mechanisms analysis. Thus far, codon usage bias in *S. agalactiae* has not been investigated in detail, and it is unclear whether there are differences in gene expression based upon selection pressures. Therefore, we studied codon usage bias of *S. agalactiae* genes using codon usage statistics, multivariate statistical techniques and correlation analysis. Moreover, we investigated the codon usage bias of *S. agalactiae* and the adaptation relationship between *S. agalactiae* and its hosts, in order to obtain clues to genetic evolution of *S. agalactiae*.

## 2. Results

### 2.1. The Synonymous Codon Usage Pattern of S. agalactiae Genomes

In this study, we compared five *S. agalactiae* strains whose complete genomes were available from the Genbank database. The average GC12% (G + C content at the first and second codon positions) and GC3% (G + C content at the third codon position) of all functional genes was 33.71% and 25.87%, respectively. The nucleotide contents at these positions also had significant fluctuations, namely, GC12% varied from 18.53% to 56.41%, GC3% varied from 12.78% to 53.57% in all functional genes in *S. agalactiae*. This suggested that the A/T nucleotide at the third (wobble) position were preferred over G/C at the wobble site.

Analysis of variance (ANOVA) analysis also revealed that C nucleotide at the wobble position was significantly different (*p* = 0.001) between the leading and the lagging strands, However, the three other nucleotides at the wobble position were not significantly different between the leading and the lagging strands (data not shown). This result suggested that although the GC content of the *S. agalactiae* genome was considerably low, mutation pressure from C content variation played a role in the formation of the nucleotide content of the *S. agalactiae* genome to a relatively small degree.

The codon usage data and relative synonymous codon usage (RSCU) values for all preferentially used codons between five datasets were listed in Table 1. All codons were all A- or U-ended with the exception of the UUG (Leu) codon (marked in bold in Table 1), this demonstrated that A/T compositional constraints might play a key role in contributing to the synonymous codon usage pattern. In addition, since these genomes showed an A + T redundancy (mean 64.24, SD 3.50), compositional limitation also influenced the codon usage pattern of *S. agalactiae*.

### 2.2. The Overall Codon Usage Pattern of S. agalactiae

**Figure 1:**
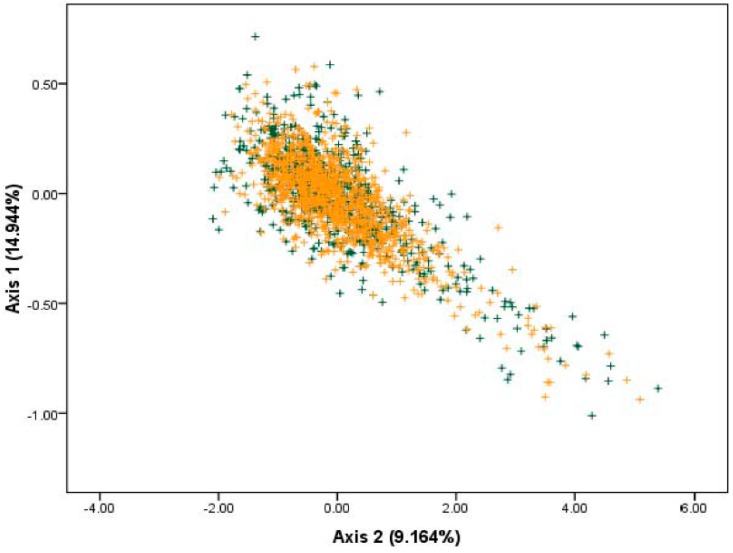
The plot of the two most dominant axes (axis 1 and axis 2) calculated from PCA according to all open reading frames (ORFs) of *S. agalactiae* genes. Genes located on the leading strand were denoted as green plus signs, whereas orange plus signs indicated lagging strand genes. Axis 1 represented the first major trend and axis 2 represented the second major trend.

The overall codon usage pattern of each gene in the *S. agalactiae* genomes was determined by PCA based on the RSCU values. From this, we could detect one major trend in the first axis, which accounted for 14.944% of the total variation, and another major trend in the second axis, which accounted for 9.164% of the total variation (Figure 1). This finding indicated that the first major axis could explain a substantial amount of variation in trends in codon usage of *S. agalactiae*, the second major axis also had an appreciable impact on total variation in codon usage pattern of *S. agalactiae* [21].

When we analyzed strand-specific mutational bias, we could find no factor influencing strand-specific mutational bias using overlay plots of both the leading strand (green plus sign) and the lagging strands (orange plus sign) (Figure 1). Therefore, strand compositional asymmetry was not the main factor in shaping the codon usage patterns in *S. agalactiae*.

The negative correlations between GC and AT skews including all three nucleotide positions as well as the first and second positions indicated that G/C and A/T composition bias had slight ability to influence the synonymous codon usage of *S. agalactiae* (Table 2). On the other hand, AT skews at the third codon position (AT3 skew) was stronger than that of mutation pressure caused by GC and AT skews at the first and second codon positions. GC3 skew was also slightly, but significantly, higher suggesting a role for GC compositional bias at the third codon position even though *S. agalactiae* has an AT-rich genome.

**Figure 2:**
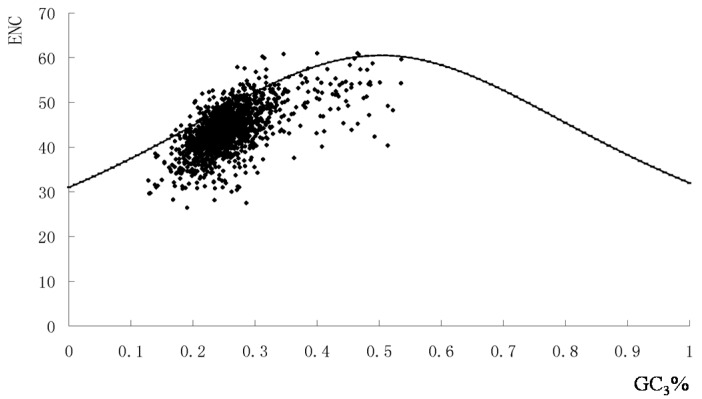
Graphs showing the relationship between effective number of codon (ENC) and the GC3% in functional genes of *S. agalactiae* genomes. The continuous curve indicated the expected codon usage if GC compositional constraints alone account for codon usage bias.

To examine this further, we plotted GC_3_% against the effective number of codons (ENC) including data from all five genomes. The majority of points with low ENC values lay below the expected curve (Figure 2). This suggested that apart from the compositional bias, mutational pressure in the codon usage pattern was the most important factor in determining codon usage bias.

The majority of points with low ENC values lay below the expected curve (Figure 2). This suggested that apart from the compositional bias, mutational pressure in the codon usage pattern was the most important factor in determining codon usage bias.

Most of the genes fell within a restricted cloud in a relatively narrow range of GC_3_ values. However, these points represented a wide range of ENC values indicating that translational selection was also responsible for codon bias among the genes. The few points that lay above the curve could be accounted for by extreme compositional constraints (Figure 2). Furthermore, the negative correlation between the ENC value and the first major variation (Axis 1) was analyzed (*r* = 0.048, *p* < 0.01). Together, these results suggested that both nucleotide composition and other evolutionary factors played roles in the overall codon usage in the *S. agalactiae* genome.

### 2.3. Using PR—2 Plots to Differentiate Replication and Transcription Associated Biases

**Figure 3:**
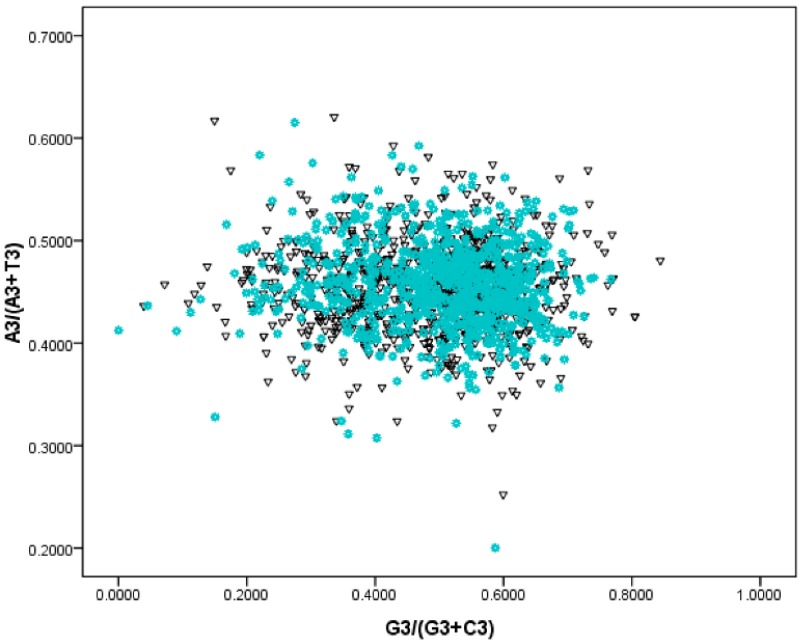
PR2—plot for *S. agalactiae* genomes. Genes located on the leading strand were denoted as black triangles, whereas blue asterisks indicated lagging strand genes.

As mentioned above, compositional bias among genes is very strong in the *S. agalactiae* genomes. These biases can be induced by many factors, such as asymmetric replication mutation pressure, asymmetric transcription/translation—associated mutation/selection pressure [12]. The G/C strand bias indicated by the horizontal axis were slightly stronger than that of the T/A (vertical axis) (Figure 3). Following the rule of PR—2 plots, the extent of replication—induced bias value was 0.322, and the transcription/translation-associated bias value was 0.013. Therefore, the replication effect on gene composition bias was much higher than that of transcription/translation effects.

### 2.4. Genetic Diversities Based on Synonymous Codon Usage by PCA

Negative correlation was found with gene length against ENC value, suggesting that gene length had little influence on codon usage. A negative correlation was also found with gene length against GC3%, codon adaptation index (CAI), the first major trend by PCA (Axis 1) and the second major trend by PCA (Axis 2) through correlation analysis. These findings suggested that gene length was not playing a major role in the case of codon usage bias in this organism.

In addition, the significant correlation between the first major trend by PCA (Axis 1) and GC3% was observed (Spearman *r* = 0.055 *, *p* < 0.05), further highlighted the fact that the nucleotide compositional constraint from mutational pressure was one factor in shaping the codon usage patterns in the *S. agalactiae* genomes.

### 2.5. Codon Adaptation Index (CAI) and Identification of Translationally Optimal Codons

**Figure 4:**
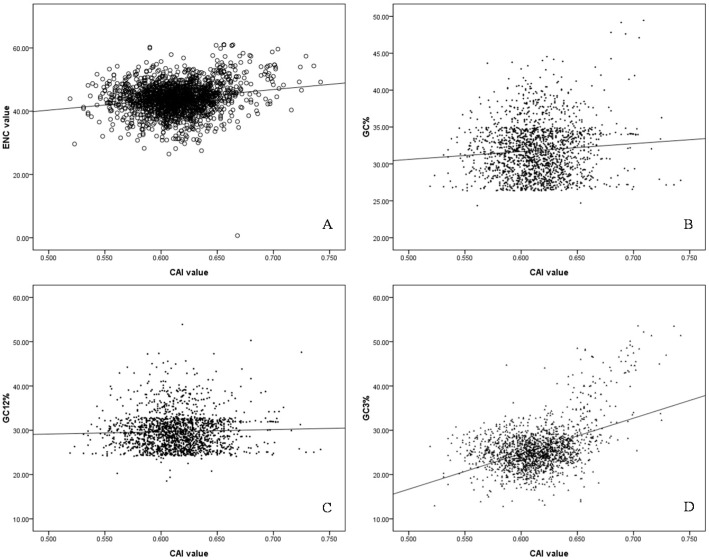
The correlation between CAI values and ENC, GC%, GC12%, GC3% values. (**A**) the correlation between CAI values and ENC values, the black dot represented the ENC value against CAI value for each gene, the black line was generated by the correlation analysis; (**B**) the correlation between CAI values and GC% values, the black dot represented the GC% value against CAI value for each gene, the black line represented linear fitting line of all data; (**C**) the correlation between CAI values and GC12% values, the black dot represented the GC12% value against CAI value for each gene, the black line represented linear fitting line of all data; (**D**) the correlation between CAI values and GC3% values, the black dot represented the GC3% value against CAI value for each gene, the black line represented linear fitting line of all data.

CAI value has the ability to predict levels of gene expression, significant correlations between the gene expression levels through CAI value and ENC, GC%, GC12% and GC3% (Spearman *r* = 0.136 **, 0.233 **, 0.056 *, 0.277 *, respectively, *p* < 0.01) were analyzed (Figure 4), and the negative correlation between the first major trend (Axis 1) and CAI was evident in *S. agalactiae* genomes (Spearman *r* = 0.014, *p* < 0.01), indicating that the higher expression genes would cluster on the extreme end of the first major trend from PCA. This demonstrated that gene expression level was the most important factor in codon usage in the *S. agalactiae* genomes. In addition, genes with higher expression levels exhibited a greater degree of codon usage bias.

In addition, we found significant correlation between the first major trend (Axis 1) on RSCU and frequency of optimal codons (Fop) value (Spearman *r* = 0.053 *, *p* < 0.01), indicating the genes variation resulted from their usage preference of optimal codons. And significant correlations between CAI value and Fop value (Spearman *r* = 0.078 *, *p* < 0.01), suggested that the high expression genes preferentially used a particular subset of optimal codons in order to enhance their translational efficiency.

Twenty-three codons were determined as the optimal codons containing 11 A-ending and 16 U-ending codons (Table 1). These optimal codons might have the ability to influence expression levels of heterologous genes in order to increase the abundance of specific proteins, which were recognized by the most abundant tRNAs. Therefore, translational selection had influenced the synonymous codon pattern of *S. agalactiae* and highly expressed genes used more optional codons frequently. Furthermore, the positive correlation between the tRNA adaptation index (tAI) values and CAI values (Spearman *r* = 0.277 *, *p* < 0.01) and Fop values (Spearman *r* = 0.455 *, *p* < 0.01) in the *S. agalactiae* genomes, reinforced the role of natural selection for efficient translation.

### 2.6. Effects of the Overall Codon Usage of Hosts on that of S. agalactiae

According to potential role of the overall codon usage pattern (D (*A*, *B*)) values reflecting the effect of overall codon usage pattern of human, tilapia, common carp, and crucian carp on that of *S. agalactiae*, the average of D (*A*, *B*) values were 0.1686, 0.1694, 0.15579, 0.1566, respectively. These low values indicated a strong independence from host pressures in the evolution of its synonymous codon usage patterns. The bacterium acted like an independent replication unit that did not need components from its host for translation unlike what a virus would require.

## 3. Discussion

The majority of codon usage variations seen in the *S. agalactiae* genome was affected by mutational pressure. In this study, a large number of points with low ENC values lie below the expected curve (ENC against GC3%), the result suggests that apart from the compositional bias, mutational pressure in codon usage pattern is the most important factor in shaping the codon usage bias of *S. agalactiae*. A significant correlation was found between the first major trend (axis 1) generated from PCA and GC3%, further highlighting that the nucleotide compositional constraint from mutational pressure is the major source of the codon usage patterns. In addition, we found that the synonymous codon usage bias in *S.agalactiae* genes was low (mean ENC = 44.06, higher than 40). The low codon bias may assist *S. agalactiae* to replicate efficiently in the host cells through affecting the cellular protein synthesis and host mRNAs degradation, this would serve to reduce competition between the bacteria and its host [21]. But low D (*A*, *B*) value suggests that *S. agalactiae* has a simple form of self-replication with a strong independence in its evolution of synonymous codon usage. Compositional bias is the other factor influencing the formation of synonymous codon usage of *S. agalactiae*, *S. agalactiae* is an A + T redundant genome with A + T content more than 50% (mean 64.24, SD 3.50), the AT richness of this genus is a distinctive feature and this is reflected in its codon usage variations. Nevertheless, GC compositional bias also accounts for the observed variations. Translational selection was also evident from the fact that codon usage in the genomes of *S. agalactiae* was driven by the tRNA aimed at enhancing translational adeptness, and stems from the observation of significant correlations between the tAI and CAI, FOP value, reinforcing the role of natural selection on translational efficiency. The same results were reported for other organisms like *Escherichia coli*, *Caenorhabditis elegans*, and *Bifidobacterium* spp. [22,23,24]. Thus, the effect of compositional constraints and translation selection played roles in shaping the codon usage fashion of this species.

CAI value is measured to predict gene expression levels. In this study, we analyzed the correlation between codon usage bias and predicted gene expression levels, and found a significant correlation. The optimal codons contained 10 A-ending and 16 U-ending codons, maybe have the ability to influence expression levels of heterologous genes in order to increase the abundance of specific protein, the same results are reported in *E. coli*, *Drosophila melanogaster*, *Caenorhabditis elegans*, *Chromohalobacter salexigens*, *Aedes aegypti* and *Anopheles gambiae* [22,23,25,26,27].

*S. agalactiae* is a rapidly evolving bacterium through phylogentic analysis, which is located on one of the longest branches of the phylogenetic tree life. Based on the biological characterizations, *S. agalactiae* have the ability to survive hostile environments and accommodate new environments or hosts after *S. agalactiae* infects its natural hosts. So, synonymous codon usage patterns of the *S. agalactiae* genome were independent of that of hosts.

## 4. Materials and Methods

### 4.1. Sequence Data

To investigate the codon usage pattern of functional genes of *S. agalactiae* isolated from human and tilapia. The five complete genomes sequence of *S. agalactiae* A909 (GenBank No. CP000114.1), NEM316 (GenBank No. AL732656.1), 2603V/R (GenBank No. AE009948.1), COH1 (GenBank No. HG939456.1), GD201008-001 (GenBank No. CP003810.1) were downloaded from the GenBank database [28]. Each gene in the leading or lagging strand was recorded to estimate the role of the strand-specific mutational bias in the formation of synonymous codon usage for *S. agalactiae*. In order to avoid errors, sequences with length less than 300 bps were excluded for this study, thus the remaining gene sequences were used for the analysis. In addition, codon usage frequencies of susceptible hosts including human (*Homo sapiens*), tilapia (*Oreochromis mossambucus*) and nonsusceptible hosts including common carp (*Cyprinius carpio*), crucian carp (*Carassius auratus*) were calculated according to the codon usage database to investigate codon adaptation to the hosts [29].

### 4.2. Codon Usage Pattern of Functional Genes of S. agalactiae

The ENC value against the GC3% (G + C content at the third codon position) is one of the best measures to predict heterogeneity from expected random codon usage of genes. ENC values range from 20 to 61. When only one codon is used for the corresponding amino acid, this value would be 20; and if all synonymous codons are used equally, it would be 61. The stronger the extent of codon preference in a gene, the lower the ENC value is [21,30,31].

### 4.3. Analysis of the Genetic Diversities of the S. agalactiae ORFs by Principle Component Analysis (PCA)

Based on the relative synonymous codon usage (RSCU) values, PCA was employed in this study to investigate the genetic diversity of *S. agalactiae* using genes larger than 300 bps, which reduced data dimensionality by performing a covariance analysis among the 59 synonymous codons.

### 4.4. Nucleotide Composition Statistics for Genes in S. agalactiae

Strand asymmetry of the nucleotide compositions exists between the leading and lagging strands. Specifically, when excess of guanine (G) bases relative to cytosine (C) exists in the leading strands, the opposite base scale exists in the lagging strands, while the characteristic is frequently accompanied by the bias of thymine (T) *versus* adenine (A). Herein, a series of GC skew and AT skew data were calculated for codons at the first and second positions and at the third position to evaluate the effects of nucleotide composition bias at different nucleotide positions on synonymous codon usage pattern [32,33].

### 4.5. Replication and Transcription Associated Biases Differentiation

Expected theoretical value is A = T and G = C in each strand if there are no strand-specific biases between the two strands of DNA to evaluate the asymmetry in mutation and/or selection between the two strands G/(G + C) value against A/(A + T) value at the 3rd codon position in all genes proposed by Lobry and Sueoka [34]. Here, we evaluated nucleotide composition bias in *S. agalactiae* genomes according to the PR2—plot method.

### 4.6. Preferred Gene Analysis by Codon Adaptation Index (CAI)

CAI is used to estimate predict levels of gene expression. The CAI value ranges from 0 to 1.0 for a gene, where a higher value is likely to indicate stronger codon usage bias and a potential higher expression level [21,35,36]. The CAI value was calculated using EMBOSS CUSP program [37].

### 4.7. Computation of tRNA Adaptation Index (tAI) and Frequency of Optimal Codons (Fop)

tRNA adaptation index (tAI) is a simple estimate of tRNA usage by the coding sequences of a genome, which represents the levels of co-adaptation between a particular codon and the corresponding tRNA pool and produces higher correlations with protein abundance than the other measures of codon bias [38]. The tAI values for the *S. agalactiae* genomes were calculated according to dos Reis *et al.* [39]. Frequency of optimal codons (Fop) is calculated using codonW program [40].

### 4.8. Estimating Effects of the Overall Codon Usage of Human and Tilapia on that of S. agalactiae

To estimate the effect of the overall codon usage of human, tilapia on that of *S.agalactiae*, a formula of D (*A*, *B*) was used to evaluate the potential role of the overall codon usage pattern of human and tilapia in the formation of codon usage of *S. agalactiae*, comparing with nonsusceptible hosts including *Cyprinius carpio and Carassius auratus*.

(1) $$R(A,B)=\frac{\sum_{i=1}^{59}a_{i}\times b_{i}}{\sqrt{\sum_{i=1}^{59}a_{i}^{2}}\times \sqrt{\sum_{i=1}^{59}b_{i}^{2}}}$$

(2) $$D(A,B)=\frac{1-D(A,B)}{2}$$

where R (*A*, *B*) is defined as a cosine value of an included angle between *A* and *B* special vectors representing the degree of similarity between *S. agalactiae* and hosts at the aspect of the overall codon usage pattern, a*_i_* is defined as the RSCU value for a specific codon in 59 synonymous codons of *S. agalactiae*, b*_i_* is termed as the RSCU value for the same codon of hosts. D (*A*, *B*) represents the potential effect of the overall codon usage of human and tilapia on that of *S. agalactiae*, and D (*A*, *B*)∈(0,1) [41]. The higher D (*A*, *B*) is, the stronger the effect of environment related synonymous codon usage patterns of hosts on that of *S. agalactiae* is.

### 4.9. Statistical Analysis

PCA was employed to analyze major trend in codon usage pattern which reduces data dimensionality by performing a covariance analysis between 59 synonymous codons [5,21,42]. Correlation analysis involved in this study was based on the Spearman’s rank correlation analysis and was performed using the SPSS program.

## 5. Conclusions

Synonymous codon usage pattern analysis has contributed to understand codon usage bias in *S. agalactiae*. Based on the biological characterizations, *S. agalactiae* have the ability to survive hostile environments and accommodate new environment or hosts. And codon usage pattern analysis is helpful to understand the evolution of *S. agalactiae* in this study.

## Figures and Tables

**Table 1:** The synonymous codon usage pattern in all open reading frames (ORFs) of *S. agalactiae*.

Amino Acid	Codon	RSCU ^a^
Ala	GCA	**1.39**
	GCC	0.44
	GCG	0.37
	GCU	**1.80**
Arg	AGA	**1.55**
	AGG	0.56
	CGA	0.72
	CGC	0.67
	CGG	0.18
	CGU	**2.32**
Asn	AAC	0.50
	AAU	**1.50**
Asp	GAC	0.43
	GAU	**1.57**
Cys	UGC	0.41
	UGU	**1.47**
Gln	CAA	**1.55**
	CAG	0.44
Glu	GAA	**1.48**
	GAG	0.52
Gly	GGA	**1.28**
	GGC	0.46
	GGG	0.47
	GGU	**1.79**
His	CAC	0.53
	CAU	**1.46**
Ile	AUA	0.55
	AUC	0.61
	AUU	**1.84**
Leu	CUA	0.73
	CUC	0.35
	CUG	0.25
	CUU	**1.22**
	UUA	**2.45**
	UUG	0.98
Lys	AAA	**1.52**
	AAG	0.48
Phe	UUC	0.45
	UUU	**1.56**
Pro	CCA	**1.80**
	CCC	0.32
	CCG	0.32
	CCU	**1.55**
Ser	AGC	0.57
	AGU	**1.43**
	UCA	**1.76**
	UCC	0.32
	UCG	0.32
	UCU	**1.61**
Thr	ACA	**1.59**
	ACC	0.48
	ACG	0.41
	ACU	**1.52**
Tyr	UAC	0.51
	UAU	**1.48**
Val	GUA	**1.12**
	GUC	0.51
	GUG	0.44
	GUU	**1.92**

^a^ Average value of RSCU in five *S. agalactiae* genome; underline represented A/U rich-ending compositional constraints.

**Table 2:** Nucleotide composition statistics for a gene population in five *S. agalactiae* genomes.

Skew	Axis 1	Axis 2
GC skew	*r* = 0.022, *p* > 0.05	*r* = 0.006, *p* > 0.05
AT skew	*r* = −0.015, *p* > 0.05	*r* = 0.01, *p* > 0.05
GC12 skew	*r* = 0.003, *p* > 0.05	*r* = 0.13, *p* > 0.05
AT12 skew	*r* = 0.016, *p* > 0.05	*r* = 0.019, *p* > 0.05
GC3 skew	*r* = 0.067 *, *p* < 0.05	*r* = 0.056 *, *p* < 0.05
AT3 skew	*r* = 0.628 **, *p* < 0.01	*r* = 0.555 *, *p* < 0.01

* Significant difference between them; ** highly significant difference between them.
